# Supplementary material for: New Aptamer/MoS2/Ni-Fe LDH Photoelectric Sensor for Bisphenol A Determination
Source: Nanomaterials (Basel). 2021 Dec 28;12(1):78. doi: 10.3390/nano12010078 (PMC8746877; doi:10.3390/nano12010078)
Supplement: Supplementary file 1 [file nanomaterials-12-00078-s001.zip › nanomaterials-1366344-supplementary.pdf]

Supplementary Materials

New Aptamer/MoS_2/Ni-Fe LDH Photoelectric Sensor for Bisphenol A Determination

Hongjie Gao, Yun He and Jiankang Liu *

The Key Laboratory of Biomedical Information Engineering, Ministry of Education, Mitochondrial Biomedical Research Institute, School of Life Science and Technology, Xi'an Jiaotong University, Xi'an 710049, China; gao317021hongjie@163.com (H.G.); y984420yuner_gao@126.com (Y.H.)

* Correspondence: slst@xjtu.edu.cn

1. EIS of all materials

EIS analysis has been frequently adopted for investigating electrode process kinetics as well as charge transfer on material surface. EIS is suggested as the efficient electron transfer rate characterization within the PEC materials. Our EIS analysis result is presented in Figure 4B. Besides, the low EIS curve curvature radius is associated with the great electron transfer rate. As for our prepared aptamer/MoS_2/LDH-5%, its curvature radius remarkably decreases relative to those for other materials. This might be related to introduction of MoS_2 that led to improved PEC properties and rapidest electron transfer rate.

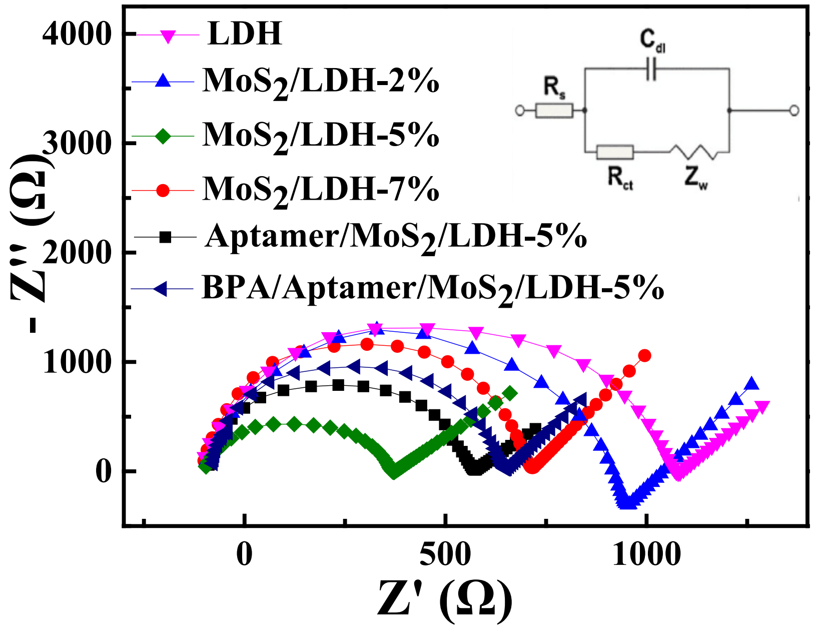

Figure S1. EIS of all materials in PBS with $Fe(CN)_6^{3+}/Fe(CN)_6^{4+}$(5 mmol L^{-1}).

The photocurrent responses of five aptamer/MoS_2/LDH-5% modified electrodes in parallel towards 50 ng L^{-1} BPA were estimated (Fig. S2A) and the stability of the aptamer/MoS_2/LDH-5% photoelectrode were evaluated by testing the photocurrent response to 50 ng L^{-1} BPA every 2 days (Fig. S2B). Data suggests that the aptamer/MoS_2/LDH-5% exhibits a good stability and reusability. The selectivity of aptamer/ MoS_2/LDH-5% was text by anti-interference experiments.

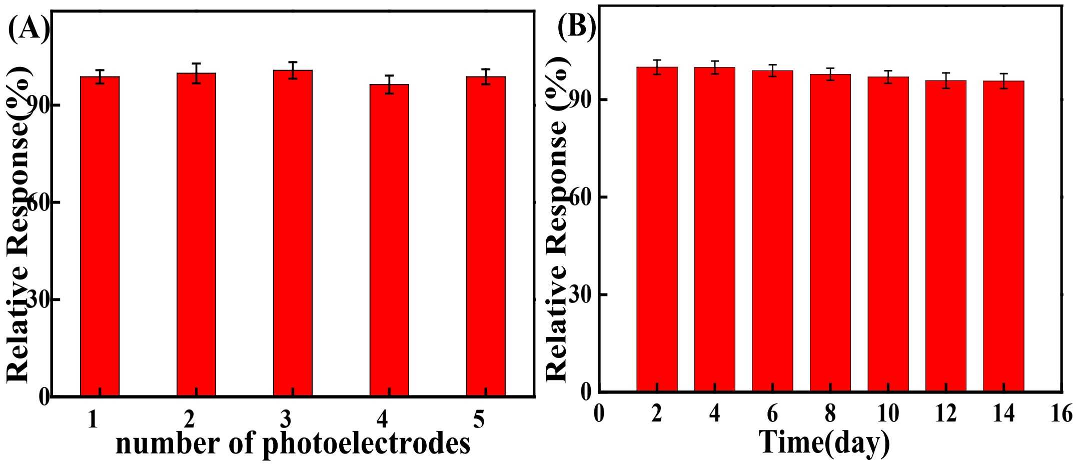

Figure S2. (A) photocurrent responses of five-paralleled aptamer/MoS_2/LDH-5% photoelectrodes with 50 ng L^{-1} BPA; (B) Stability tests of PEC base on aptamer/MoS_2/LDH-5% towards 50 ng L^{-1} BPA.

Table S1. Comparison of different ways for determination BPA.

Method	Materials	Linear Range (ng.L^{-1})	LOD (ng.L^{-1})	Ref
Amperometry	hydroxylated multi-walled carbon nanotubes	1.00×10^{4}-2.51×10^{4}	8.10×10^{2}	1
Differential pulse voltammetry	gold nanoclusters	1.14×10^{4}-1.26×10^{7}	2.74×10^{2}	2
PEC aptasensor	Bi/CN	2.00×10^{-2}-2.00×10^{3}	6.70×10^{-3}	3
PEC aptasensor	g-C_3N_4/CuO	0.02-10<br>50-1200	6.20×10^{-3}	4
PEC aptasensor	AuNPs /g-C3N4	2.28×10^{1}-2.28×10^{5}	6.85	5
PEC aptasensor	MoS_2/Ni-Fe LDH	0.05-10<br>50-4.00×10^{3}	5.20×10^{-3}	This Work

Table S2. PEC aptasensor of BPA in real river water samples

sample	Added ($ng \cdot L^{-1}$)	Found ($ng \cdot L^{-1}$)	Recovery (%)	RSD (%)	HPCL ($ng \cdot L^{-1}$)
1	5.00	5.02-4.93	100.40-98.60	3.06	4.79
2	50.00	50.12-49.91	100.24-99.82	2.36	49.97
3	100.00	100.22-99.95	100.22-99.95	3.09	99.98
4	500.00	500.27-499.95	100.05-99.99	2.91	499.83
5	1000.00	1005.26-998.41	100.53-99.84	3.24	999.07

References

1. Cosio, M.S.; Pellicanò, A.; Brunetti. B.; Fuenmayor, C.A. A simple hydroxylated multi-walled carbon nanotubes modified glassy carbon electrode for rapid amperometric detection of bisphenol A. *Sensor. Actuat. B-Chem.* **2017**, *246*, 673-679.

2. Chen, W.Y.; Mei, L.P.; Feng, J.J.; Yuan, T.; Wang, A.J.; Yu, H.Y. Electrochemical determination of bisphenol A with a glassy carbon electrode modified with gold nanodendrites. *Microchim. Acta.* **2015**, *182*, 703-709.

3. Yan, P.C.; Mo, Z.; Xu, L.; Pang, J.Y.; Qian, J.C.; Zhao, L.; Zhang, J.M.; Chen, J.P.; Li, H.N. Plasmonic Bi microspheres doped carbon nitride heterojunction: Intensive photoelectrochemical aptasensor for bisphenol A. *Electrochim. Acta.* **2019**, *319*, 10-17

4. Yang, L.Q.; Zhao, Z.J.; Hu, J.; Wang, H.B.; Dong, J.F.; Wan, X.; Cai, Z.Y.; Li, M.Y. Copper Oxide Nanoparticles with Graphitic Carbon Nitride for Ultrasensitive Photoelectrochemical Aptasensor of Bisphenol A. *Electroanal.* **2020**, *32*, 1651-1658

5. Deiminiat, B.; Gholam, H.R. A novel visible light photoelectrochemical aptasensor for determination of bisphenol A based on surface plasmon resonance of gold nanoparticles activated g-C3N4 nanosheets. *J. Electroanal. Chem.* **2021**, *886*, 115122.
